# Supplementary material for: Rebound activation of 5-HT neurons following SSRI discontinuation
Source: Neuropsychopharmacology. 2024 Apr 12;49(10):1580–9. doi: 10.1038/s41386-024-01857-8 (PMC11319583; doi:10.1038/s41386-024-01857-8)
Supplement: Supplementary file 1 — Supplementary Tables [file 41386_2024_1857_MOESM1_ESM.pdf]

Table S1 Effect of 2 days discontinuation from 12 days once-daily paroxetine (10 mg/kg) on regional brain tissue levels of DA and DOPAC. Mean± SEM values are shown (nmol/mg tissue), 8 mice/group. Repeated measures ANOVA with Bonferroni's test. DA: effect of treatment $F(2,21)=0.844$, $p=0.433$, effect of region $F(3,63)=74.49$, $p<0.001$, treatment*region interaction $F(6,63)=0.263$, $p=0.953$. DOPAC: effect of treatment $F(2,21)=0.364$, $p=0.696$, effect of region $F(3,63)=51.21$, $p<0.001$, treatment*region interaction $F(6,63)=0.256$, $p=0.956$.

	**Treatment**		
**Brain region**	**SAL**	**CON**	**DIS2**
DA			
Midbrain	0.111 ± 0.016	0.135 ± 0.014	0.134 ± 0.025
Hippocampus	0.132 ± 0.021	0.162 ± 0.032	0.148 ± 0.029
Striatum	3.025 ± 0.529	3.448 ± 0.415	3.514 ± 0.610
Frontal cortex	0.657 ± 0.114	0.921 ± 0.140	1.219 ± 0.493
DOPAC			
Midbrain	0.030 ± 0.002	0.026 ± 0.003	0.031 ± 0.006
Hippocampus	0.020 ± 0.004	0.020 ± 0.007	0.021 ± 0.002
Striatum	0.289 ± 0.067	0.330 ± 0.053	0.309 ± 0.051
Frontal cortex	0.120 ± 0.022	0.135 ± 0.011	0.174 ± 0.047

Table S2 Effect of 5 days discontinuation from 12 days once-daily paroxetine (10 mg/kg) on regional brain tissue levels of DA and DOPAC. Mean± SEM values are shown (nmol/mg tissue) with 9 mice/group. Repeated measures ANOVA with Bonferroni's test. N=9/group. DA: effect of treatment $F(2,24)=2.325$, $p=0.109$, effect of region $F(3,72)=15.00$, $p<0.001$, treatment*region interaction $F(6,72)=0.539$, $p=0.708$. DOPAC: effect of treatment $F(2,24)=2.483$, $p=0.092$, effect of region $F(6,72)=71.07$, $p<0.001$, treatment*region interaction $F(6,72)=0.827$, $p=0.554$.

	Treatment		
**Brain region**	**SAL**	**CON**	**DIS5**
DA			
Midbrain	0.048 ± 0.010	0.063 ± 0.15	0.067 ± 0.013
Hippocampus	0.074 ± 0.025	0.084 ± 0.025	0.163 ± 0.047
Striatum	0.975 ± 0.271	1.935 ± 0.399	2.190 ± 0.498
Frontal cortex	0.419 ± 0.115	0.423 ± 0.142	0.613 ± 0.128
DOPAC			
Midbrain	0.025 ± 0.004	0.028 ± 0.004	0.056 ± 0.034
Hippocampus	0.013 ± 0.004	0.020 ± 0.003	0.033 ± 0.008
Striatum	0.291 ± 0.063	0.342 ± 0.058	0.391 ± 0.044
Frontal cortex	0.073 ± 0.017	0.086 ± 0.023	0.128 ± 0.025

Table S3 Effect of 2 and 5 days of paroxetine discontinuation on mRNA expression of 5-HT receptor subtypes in mouse midbrain raphe region and prefrontal cortex. Mice were injected once daily for 12 days with either saline (SAL) or 10 mg/kg paroxetine (CON), with the latter discontinued for either 2 (DIS 2) or 5 (DIS 5) days. Values for mRNA (mean ± SEM) are relative to house-keeping genes, n=6-8/group. One-way ANOVA - post-hoc comparisons, # $p<0.05$ CON vs DIS, * $p<0.01$ CON vs DIS.

	**Discontinuation day 2**			
	**SAL**	**CON**	**DIS 2**	**ANOVA**
Midbrain region				
5-HT$_{1A}$	1.03 ± 0.10	1.34 ± 0.13	0.99 ± 0.10#	$F(2,21)=2.949$, $p=0.0444$
5-HT$_{1B}$	1.05 ± 0.13	1.23 ± 0.20	0.88 ± 0.14	$F(2,21)=1.417$, $p=0.2835$
PFC				
5-HT$_{1A}$	1.02 ± 0.08	1.10 ± 0.10	0.76 ± 0.07* #	$F(2,21)=4.529$, $p=0.0232$
5-HT$_{1B}$	1.05 ± 0.11	1.88 ± 0.42	1.44 ± 0.23	$F(2,21)=2.098$, $p=0.1477$
5-HT$_{2A}$	1.01 ± 0.06	1.03 ± 0.09	0.93 ± 0.10	$F(2,21)=0.3668$ $p=0.6973$
5-HT$_{4}$	1.08 ± 0.16	0.94 ± 0.09	1.81 ± 0.64	$F(2,21)=1.264$ $p=0.3052$

	**Discontinuation day 5**			
	**SAL**	**CON**	**DIS 5**	**ANOVA**
Midbrain region				
5-HT$_{1A}$	1.03 ± 0.12	0.85 ± 0.06	1.07 ± 0.26	$F(2,14)=1.281$ $p=0.3084$
5-HT$_{1B}$	1.02 ± 0.08	1.10 ± 0.17	0.85 ± 0.06	$F(2,15)=1.177$ $p=0.3352$
PFC				
5-HT$_{1A}$	1.02 ± 0.08	1.15 ± 0.07	1.02 ± 0.08	$F(2,15)=0.8851$ $p=0.4332$
5-HT$_{1B}$	1.01 ± 0.07	1.06 ± 0.09	1.17 ± 0.17	$F(2,15)=0.4864$ $p=0.6242$
5-HT$_{2A}$	1.02 ± 0.07	1.01 ± 0.08	1.18 ± 0.16	$F(2,15)=0.7104$ $p=0.5072$
5-HT$_{4}$	1.00 ± 0.05	0.92 ± 0.07	0.86 ± 0.10	$F(2,14)=0.8139$ $p=0.4630$
